# Dynamic-Vision-Based Force Measurements Using Convolutional Recurrent Neural Networks

**DOI:** 10.3390/s20164469

**Published:** 2020-08-10

**Authors:** Fariborz Baghaei Naeini, Dimitrios Makris, Dongming Gan, Yahya Zweiri

**Affiliations:** 1 Faculty of Science, Engineering and Computing, London SW15 3DW, UK; D.Makris@kingston.ac.uk (D.M.); Y.Zweiri@Kingston.ac.uk (Y.Z.); 2 School of Engineering Technology, Purdue University, West Lafayette, IN 47907, USA; dgan@purdue.edu; 3 Khalifa University Center for Autonomous Robotic Systems (KUCARS), Khalifa University, Abu Dhabi P.O. Box 127788, UAE

**Keywords:** vision-based measurements, dynamic force estimation, dynamic vision sensor, LSTM, neuromorphic sensor, even-based sensor

## Abstract

In this paper, a novel dynamic Vision-Based Measurement method is proposed to measure contact force independent of the object sizes. A neuromorphic camera (Dynamic Vision Sensor) is utilizused to observe intensity changes within the silicone membrane where the object is in contact. Three deep Long Short-Term Memory neural networks combined with convolutional layers are developed and implemented to estimate the contact force from intensity changes over time. Thirty-five experiments are conducted using three objects with different sizes to validate the proposed approach. We demonstrate that the networks with memory gates are robust against variable contact sizes as the networks learn object sizes in the early stage of a grasp. Moreover, spatial and temporal features enable the sensor to estimate the contact force every 10 ms accurately. The results are promising with Mean Squared Error of less than 0.1 N for grasping and holding contact force using leave-one-out cross-validation method.

## 1. Introduction

Tactile sensors are developed to acquire physical properties of the contact area between the sensor and environment. Similar to human skin receptors, tactile sensors are capable of measuringcan measure physical properties such as position, force, torque and temperature in the contact point. However, industrial applications require different specifications for the sensor in terms of resolution, latency, and accuracy based on the functionality of the system. Thus, researchers utilizuse a variety of measurements methods to tackle sensing challenges and overcome limitations of the sensors for diverse real-world applications [1].

The rise of robotic systems in automation increases the popularity of tactile sensors for the safe human robot interaction [2], soft robotics [3], and object recognition [4]. Different types of tactile sensing methods for dexterous robot hands are reviewed in [5], which shows the advantages of optical tactile sensors in terms of high spatial resolution, sensitivity, repeatability, and immunity to electromagnetic disturbances. Optical tactile sensors aim to extract tactile information by emitting light waves and study behaviourbehavior of back-scattered waves in a flexible membrane (fingertip). Use of different materials within the flexible membrane allows optical tactile sensors to achieve high sensitivity as illustrated in [6]. Furthermore, multi-modal optical tactile sensors combined with electrical tactile sensors demonstrate a successful application for materials classification and proximity range detection [7].

Vision-based sensors are a subcategory of optical sensors that utilizuse cameras and image processing techniques for measurement and sensing purposes. Advancement in camera technology, machine vision techniques, and processing power, increase the number of Vision-Based Measurement (VBM) systems significantly [8]. The fundamental approach of VBM sensors for tactile sensing is to monitor the contact area by camera and acquire information about physical properties as well as objects characteristics. For example, multiple Kalman filter is considered to observe the contact area deformation to estimate the contact force continuously [9]. Another approach in [10] proposed a technique to estimate the contact force from frustrated total internal reflection using a camera. One of the most popular designs is to attach markers or pins on the elastomer surface which can be tracked by computer vision algorithms to determine the contact area properties reviewed comprehensively in [11]. UtilizationUse of machine learning techniques have recently become popular in this field, as they significantly improve the accuracy of VBM sensors. However, limitations of vision sensors such as conventional cameras with limited sampling rate, high power consumption and low dynamic range that restrict the sensors performance.

A new biologically inspired type of cameras, neuromorphic vision sensors, use a differential approach to acquire information from the scene. Neuromorphic (Event-based) vision sensors capture intensity changes in a binary format at every pixel (events), timestamped using microsecond resolution, rather than absolute intensity values of all pixels at fixed frame-rateframerate. The main advantages of the neuromorphic sensors like like the Dynamic Vision Sensor (DVS) against the conventional cameras are high temporal resolution, high dynamic range and low power consumption [12].

In [13], we proposed a dynamic-vision-based tactile sensor to estimate the contact force for objects with the same size during the grasping and releasing phases using Time Delay Neural Network (TDNN). However, objects with different size have a variant contact area with the silicone membrane which requires a complex dynamic method to relate events and force measurements at each time stamp. In this paper, we refer the networks without memory and with memory as static and dynamic networks, respectively. To overcome limitations of the sensor for variant object sizes, we propose a novel method to estimate the contact force dynamically by providing memory to the sensor using different Long -Short -Term Memory (LSTM) architectures. Thus, the sensor identifies the contact size from the early stage of a grasp and estimate the contact force relatively at each timestamp.

The main contribution of this paper is to propose a novel technique to measure force for objects with different sizes using neuromorphic vision sensors. The proposed sensor observes the silicone membrane deformation in the contact area to estimate the force. Three LSTM-based deep learning models are developed, and the results are compared for dynamic force measurements. The main advantage of our dynamic method is that the history of changes in the contact area are taken into account for the force measurements at each timestamp. Therefore, the sensor is capable of measuring force continuously from start of grasp until release of the object by considering the history of contact at each pixel. This approach enables the sensor to estimate the contact force accurately even for the holding phase whereas a slight vibration occurs. Moreover, the sensor latency is reduced to 10 ms (one frame) due to the networks architectures which is 52% less compared to our previous work using Time Delayed Neural Networks (TDNN) with 21 ms delay [13]. The main application of the proposed sensor is to estimate the applied force while grasping hard objects with a flat surface repeatedly.

This paper is organized as follows: Related work is reviewed in Section 2. The proposed neuromorphic vision-based tactile sensor is described in Section 3. Recurrent deep learning methods for the force estimation are proposed in Section 4. The validation process and results are discussed in Section 5 and Section 6 respectively. Finally, conclusions and future work are presented in Section 7.

## 2. Related Work

The main principle of vision-based tactile sensors is to characterize deformation of flexible fingertip in order to acquire measurements and detect slippage in the contact point. Three main techniques are developed to acquire information from the contact area: (i) Light- conductive plate; (ii) Reflective membrane; (iii) Marker-based membrane. Light-conductive tactile sensors emit light to the contact area and the camera observers light intensity changes to acquire contact position and measure force [14]. Reflective membrane sensors consider a reflective flexible material to observe changes in the shape of membrane which enable the sensor to measure orientation as well as texture of the object [15].

Marker-based tactile sensors consider markers or pins inside the elastic fingertip and correlate displacement of the markers to the contact area properties. One of the earliest works [16], presented a technique to approximate the shape of contact area from markers displacement. The markers are selected to provide a high level of contrast with background. Therefore, markers are easily tracked by a simple intensity threshold at each frame. A similar approach is proposed in [17] whereas a finger-shape sensor is designed with two layers of markers with different colors to track the markers based on color measurements for blue and red channels. The proposed sensor measures force magnitudes in three dimensions and is suitable for a low range of the contact force with small contact areas. Both works have considerable speed limitations due to the sampling rate of the camera (30 fps) and processing time.

Recent vision-based sensors attempt to utilizuse advanced computer vision and machine learning techniques to increase the sensor capabilities. For instance, a multi-task vision based tactile sensor (GelSight) is developed to estimate the contact force and detect object slippage [15] using Convolutional Neural Networks (CNN). A pretrained CNN network (VGG-16) on ImageNet is used for transfer learning in order to estimate 3D force vector and torque over the z-axis. However, the results indicate that CNN networks cannot be generalized well on GelSight images as different contact geometries generate various features in the images for the similar amount of force.

On the other hand, dynamic (recurrent) networks benefit from time-dimension features in measurements. In [18], a dynamic network is considered to estimate object hardness independent from shape using GelSight. The sequences of images are captured in the loading phase and subtracted from the first frame. In other words, intensity changes are extracted by subtracting consecutive frames. Then, a CNNLSTM network is trained on sequences of subtracted images considering only loading phase. The dynamic network provides capability to the sensor to deal with shape-independent objects for the hardness estimation after a complete loading phase. Even though the changes in intensity obtained by subtracting frames, conventional cameras still suffer from low sampling rate as well as low dynamic range which limit the sensor performance. Moreover, LSTM-based networks are developed in [19] to estimate the contact force from sequential images on the soft objects. Similarly, CNNLSTM and 3D CNN networks are implemented in [20] to predict physical interactive force between to objects from a video. Also, in [21] CNNLSTM networks are utilizused to estimatedestimate the contact force with Mean Squared Error (MSE) of 0.13 N from video.

In [22,23] a Finite Element Model (FEM) is considered to generate groundtruthground truth and acquire the contact force distribution. The main approach utilizused an optical-flow based-based tracking algorithm is considered to create a feature vector which is correlated tocorrelated with the 3D reconstruction of the contact force by neural networks with very high accuracy for an object with the same size and shape. Optical-flow features are extracted and considered to be multiple inputs into a static deep neural network model in order toto estimate the contact force. Although the optical- flow features are used for training the network, still the network is static and cannot relate force features continuously in a dynamic manner. Conversely, an inverse Finite Element Model (iFEM) is considered in [24] to reconstruct the contact force distribution in 3D. The experiments are performed with a single object of known geometry and the normal contact force is varied between 3–4 N.

Besides different algorithms and techniques in the vision-based tactile sensing, other factors such as fingertip material and pins design affect the sensing performance significantly. Ward-Cherrier et al. [3] designed a wide range of bio-inspired and 3D-printed fingertips (TacTip) with various specifications to localize objects with less than 0.2 mm error based on pins displacements. The experiments on different fingertips show that the pins specifications have a significant impact on the tracking algorithm and the sensor accuracy. Table 1 compares state-of-the-art vision-based tactile sensors for different applications.

Neuromorphic vision-based tactile sensing is relatively a new research field which aims to utilizuse event-based cameras to acquire physical properties in the contact area. In the earliest work [25], we proposed a method to utilizuse an event-based camera (Dynamic Vision Sensor) in order to detect incipient slippage using traditional image processing techniques. Similarly, new approaches for slip detection with the DVS are investigated in [26,27,28]. In another work, we proposed a novel framework based on the Dynamic Vision Sensor (DVS) to acquire force magnitude and classify materials in a grasp [13]. Two machine learning methods, TDNN and Gaussian Process (GP), were implemented to estimate the contact force for the objects with same shape and size.

Remarkable progress has been made in vision-based tactile sensors in terms of accuracy and resolution. Nevertheless, most of the previous work considers conventional cameras and static approaches to provide the contact force measurements. CNN-based methods like [15] have shown deficiency of static networks performance for objects with different geometry while dynamic networks in [18] can handle different shapes. Even though silicone material provides a physical memory to the system, non-recurrent (static) networks suffer from time-related variables while the dynamic networks adopt the measurements based on the previous sequences.

## 3. Neuromorphic Vision-Based Tactile Sensor

**Figure 1:**
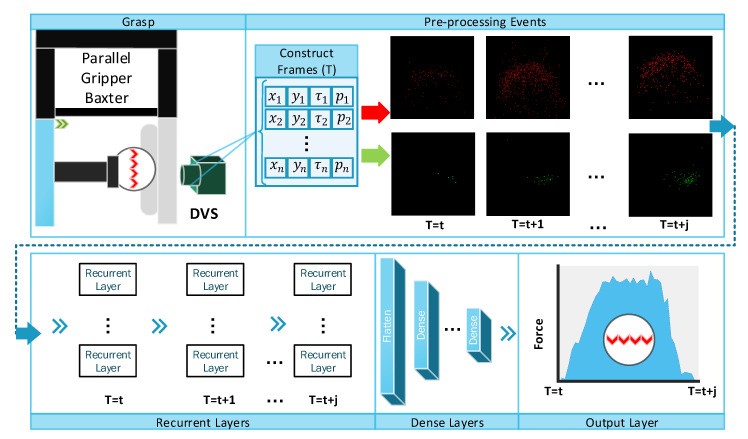
Schematic diagram of the proposed dynamic sensor for the continuous force measurements in a grasp.

The main limitations of vision-based tactile sensors are low sampling rate and high power consumption compared to other types of tactile sensors. UtilizationUse of event-based cameras for tactile sensing applications provides a much higher sampling rate as well as significant reduction of power consumption. Neuromorphic vision sensors have become significantly popular recently and introduce a paradigm in computer vision applications for instrumentation and measurements [29,30]. The Dynamic Vision Sensor (DVS) that is used in this paper is one of the well-known neuromorphic cameras. The main advantages of the DVS against standard cameras are: (i) high time resolution of few a few microseconds (average of 14 μs); (ii) high dynamic range of 120 dB; (iii) low power consumption of 5–14 mW [12]. The camera has a dimension of H 40 × W 60 × D 25 millimetre. Event-based applications consider two main approach to process events [31]: (i) event-by-event; (ii) event groups (event frame). The former approach processes events individually over time, while the latter integrates events over a time window to create a frame. Constructing frames from the events reduces the memory requirements which has been used in events compression techniques [32,33]. In this paper, we consider constructing frames from events in order toto estimate the contact force in a time window. The pipeline of the proposed dynamic-vision-based tactile sensing is demonstrated in Figure 1, whereas a neuromorphic camera (Dynamic Vision Sensor) is used to capture events. Firstly, sequential frames are constructed from events and then processed by Recurrent Neural Network (RNN) to estimate the contact force dynamically. Each stage of the proposed framework is discussed in the following sections.

### Construct Frames

The output of the DVS is a stream of events, each characterized by position, timestamp and polarity $\left(x_{k},y_{k},\tau_{k},p_{k}\right)$ whereas *k* is a counter for events and $p_{k}$ represents polarity of the pixel. Polarity is defined in a binary format which is either 0 or 1 for negative and positive polarities respectively, as shown in Figure 1. In [13], the events are accumulated over time without consideration of their position information to estimate the contact force. In this work, an event- framing technique is considered to convert raw events into sequential frames.

To process events as a group, events are accumulated within a time window (*T*). The time window is selected based on the DVS threshold, application speed and noise. Positive and negative polarities (*p*) represent a decrease or increase of the contact forceforce, respectively. Therefore, the accumulation of events are performed separately over two channels. Inspired by [34], we mathematically formulate the framing process in Equation (1), whereas $X_{t}$ represents histogram of events for different polarities p={0,1}, Kronecker delta function and rectangle function are denoted as δ and rect respectively. Timestamp of each event is represented as $\tau_{k}$ whereas *k* indicates the event index.

(1) $$X_{t}\left(x,y,p\right)=\sum_{\forall k}\mathrm{rect}\left(\frac{\tau_{k}}{T}-0.5-t\right)\;\delta_{xx_{k}}\;\delta_{yy_{k}}\;\delta_{pp_{k}}$$

Each experiment is converted to a number ofseveral sequential frames each of which represents intensity changes within a time window. The experiments are slightly varied in the length and empty frames are added to equalize the number of frames per experiment.

## 4. Dynamic Force Estimation

A dynamic sensor considers changes in measurements rather than their absolute values. Similarly, the DVS captures intensity changes and the history of each pixel is required to relate the force measurements to the triggered events at each frame. Therefore, RNNs are appropriate method to estimate the contact force based on history of frames over time. The main advantage of RNNs is an internal state that enables the network to capture sequential dependencies between variables over time. The major problem of basic RNN is the vanishing gradient whereas the network fails to learn long dependencies and the gradient decent stops to converge. To solve this problem, LSTM and Gated Recurrent Units (GRU) were introduced to control memory states [35]. We propose three architectures by combining LSTM layers with convolutional and dense layers to estimate the contact force.

### 4.1. Long Short-Term Memory Units

LSTM networks have made a significant breakthrough in time-series applications such as speech recognition and action recognition. A typical LSTM unit includes input gate, forget gate and output gate which allows the network to forget unnecessary dependencies to prevent vanishing gradients. Suppose that $X_{t}$ is the input image of the contact area to the LSTM unit and $c_{t}$ is a memory cell that accumulates states at each time.

For every timestamp, input or update gates $i_{t}$ will be activated and control the forget gate ($f_{t}$). Then, the forget gate decides the remaining images in the memory cell ($c_{t}$). Afterwards, the output gate ($o_{t}$) controls the use of images from the final state of LSTM ($h_{t}$). The forget gate and final state of the LSTM cell is initialized as zero for the first step. The controlling process of multiple gates allows the LSTM unit to be robust against the vanishing gradient problem to capture dependencies between the contact force and constructed frames. The main equations of an LSTM unit are presented in Equation (2) whereas sig is the activation function and the Hadamard product is denoted as ∘. Two matrices for inputs weights and recurrent connections are presented as *W* and *U* respectively. The initial value of *c* and *h* are defined as zero for the first step. The output information is denoted as *o* and the sigmoid function presented as sig.

(2a) $$f_{t}=sig\left(W_{f}X_{t}+U_{f}h_{t}-1+b_{f}\right)$$

(2b) $$i_{t}=sig\left(W_{i}X_{t}+U_{i}h_{t}-1+b_{i}\right)$$

(2c) $$o_{t}=sig\left(W_{0}X_{t}+U_{0}h_{t}-1+b_{o}\right)$$

(2d) $$c_{t}=f_{t}\circ c_{t-1}+i_{t}\circ sig\left(W_{c}X_{t}+U_{c}h_{t}-1+b_{c}\right)$$

(2e) $$h_{t}=o_{t}\circ sig\left(c_{t}\right)$$

The subscripts of the weight matrices indicate the input gate *i*, the forget gate *f*, the memory cell *c* or the output gate *o* while biases for each gate are presented by *b*. Often, LSTM gate’s activation functions are considered to be either sigmoid or hyperbolic tangent function. All the gates including forget Equation (2a), input Equation (2b), output gates Equation (2c) and memory cell Equation (2d) are dot-products of the weights matrices with hidden states. In fact, LSTM cell operates on the vectors and disregard spatial information of inputs and hidden states.

A single LSTM unit is sufficient for basic applications whereas the input and output relationships are not highly non-linear. On the other hand, applications with a high degree of non-linearity require further learnable parameters and multiple hidden layers to model behaviourbehavior of variables. Stacking LSTM units adds further learnable parameters and enables the network to model very complex relationships between the triggered events and the contact force with consideration of the silicone deformation. The optimal number of hidden layers and LSTM cells requires torequires to be tuned by trial and error.

### 4.2. Convolutional Long- Short Short-Term Memory Layers

Convolutional LSTM (ConvLSTM) networks are relatively new modified version of LSTMs that can capture spatial-temporal dependencies. ConvLSTM is proposed in [36], to forecast weather where the spatial-temporal dependencies are significantly important. Mathematically, the main difference of ConvLSTM layers is to replace multiplication operations with convolution denoted as (*) for controlling the gates as shown in Equation (3).

(3a) $$f_{t}=sig\left(W_{f}*X_{t}+U_{f}*h_{t}-1+b_{f}\right)$$

(3b) $$i_{t}=sig\left(W_{i}*X_{t}+U_{i}*h_{t}-1+b_{i}\right)$$

(3c) $$o_{t}=sig\left(W_{0}*X_{t}+U_{0}*h_{t}-1+b_{0}\right)$$

(3d) $$c_{t}=f_{t}\circ c_{t-1}+i_{t}\circ sig\left(W_{c}*X_{t}+U_{c}*h_{t}-1+b_{c}\right)$$

(3e) $$h_{t}=o_{t}\circ sig\left(c_{t}\right)$$

In opposed to LSTM layers, ConvLSTM layers maintain both spatial and temporal information of each frame. Due to the non-linearity of silicone material, spatial and temporal features need to be considered for estimation of the contact force during different phases.

### 4.3. Convolutional Layers with LSTM

Convolutional Neural Networks (CNNs) are designed to extract both spatial and temporal features in the image by applying convolution operations of filters in a certain window size. CNN networks have achieved significant success in different applications such as AlexNet [37] for image classification task which made CNNs a golden standard in modern computer vision. On the other hand, time-dependent applications such as video classification consider an architecture with combination of CNNs and RNNs architectures [38]. As triggered events are accumulated in a frame, CNNs are used to extract features of each frame to correlate accumulation of events with force values. Afterwards, the output of CNN layers are valid at each frame and the network needs to correlate the outputs temporally over time. Therefore, LSTM layers are considered after CNN layers to provide temporal memory for the extracted features (CNNLSTM). In the final layers, the output of LSTM is connected to the dense layers to estimate the contact force dynamically for objects with different sizes.

The main difference between CNNLSTM and ConvLSTM architectures is the order of performing convolution operations on the constructed frames. In CNNLSTM, convolution operation applies on the frames to extract features into a 1D vector which is followed by LSTM units to model extracted features temporally over time. On the other side, ConvLSTM operates convolution within the LSTM gates which maintains the two dimensiondimensions of the input to capture both spatial and temporal information of the constructed frames.

## 5. Experiments

This section presents detailed information of the experimental setup, the pre-processing stage and the networks implementation.

### 5.1. Experimental Setup

**Figure 2:**
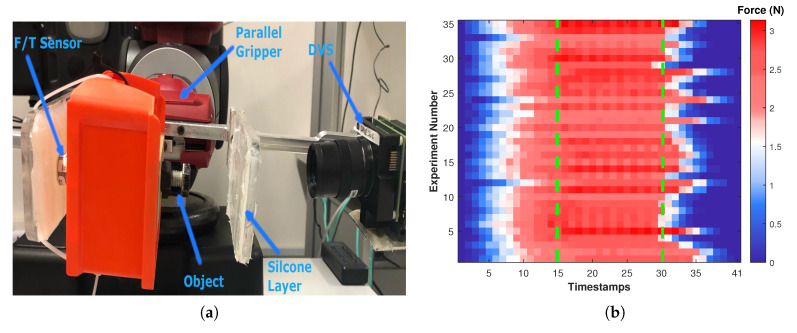
(**a**) Experimental setup to perform experiments using a Force/Torque (F/T) sensor and a DVS on the Baxter parallel gripper. (**b**) Each row shows one experiment over 41 timestamps. The colorur represents the force values (N) from low contact force (blue), medium contact force (white), and high contact force (red). The green dotted lines differentiate between the grasping, holding and releasing phases.

The experimental setup includes an ATI F/T sensor (Nano17), a DVS sensor, and a Transparent 3D printed plane (static plane) for the Baxter robot which is covered by a silicone layer as shown in Figure 2a. The silicone material has 50 shore hardness with approximately 0.5 mm thickness. Please note that the choice of the silicone with different elasticity affects the sensors sensitivity. The right plane of gripper remains static while the left plane (dynamic plane) moves to apply pressure on the silicone layer. As the silicone is not covered evenly, the object is centered to the plane to minimise effect of the silicone elasticity variations in the experiments.

For each object size, the gripper and the force sensor are calibrated first and the same process of closing and opening the grippers are followed. Although the experiments are performed with the same configuration, the contact force values and experiments duration are slightly varied due to the silicone elasticity, controllers delay, and measurements uncertainty. As the sensor estimates the contact force continuously, each experiment is divided into the grasping, holding and releasing phases and the sensor performance for each phase is investigated (Figure 2b). To evaluate the sensor performance, MSE is calculated based on based on the difference between the force sensor measurements and the predictions. Thirty-five experiments are performed on three bolts with size of 8 mm, 12 mm, 16 mm.

In each experiment, the contact force starts from zero and reaches the maximum of 3.12 N during the holding phase. The releasing phase is highly non-uniform across experiments due to the elasticity of the silicone membrane.

### 5.2. Pre-Processing

The framing process accumulates events in two different channels, as described in Section 3. The time window is selected as T=10 ms, which ensures that sufficient number of events are accumulated in frames. Furthermore, the frames are cropped based on the largest contact object contact area from 240 × 180 to 115 × 115 to reduce the memory requirements. The thresholds of positive and negative polarity are tuned to reduce noise levels. The maximum number of accumulated positive and negative polarity events across all experiments are considered in all experiments to avoid saturation in the constructed frames, i.e., their pixel values do not exceed the maximum 8-bit range. Thus, a weight function is applied to normalize the value of each pixel in two channels. As implementation of RNN requires a fixed size of sequences, maximum length is considered to be a baseline and other experiments are zero-padded to this length (410 ms).

### 5.3. Networks Implementation

The proposed networks are tuned based on trial and error considering various hyper-parameters including number of hidden layers, filters and dropout rate. The networks are designed in Keras framework [39] and trained by using NVIDIA GTX 1080 Graphical Processing Unit (GPU). LSTM cells are initialized with random orthogonal matrices which improves the robustness of LSTM layers to prevent vanishing gradient [40]. The loss function is considered to be the Mean Squared Error (MSE) to train all the networks. Adam optimizer is used for optimizing the loss function with learning rate of 0.001 and the coefficients of moving average beta1 and beta2 are set to 0.9 and 0.999 respectively.

The number of layers and network hyper-parameters are chosen experimentally. The frames represent events over a short period of time which reduce the number of features in each frame. On the other hand, each event in the frame is related to the contact force directly. Therefore, convolutional layers are designed with three filters in size of 3 × 3 and zero padding in each layer. Furthermore, drop out layers are used in the first three layers with rate of 0.4 to prevent over-fitting on the training set.

The data is split into three sets of training (31), validation (3), and test (1) sets. Then, leave-one-out technique is implemented to repeat the training and testing of the network 35 times. Early stopping technique is used to stop the training for each fold after 20 iterations without improvement of accuracy on the validation set. To select the validation set, a random experiment is picked up from each size to ensure all object sizes are included. For the final results, error of each network is averaged over all folds to select the best architecture.

## 6. Results and Discussion

**Figure 3:**
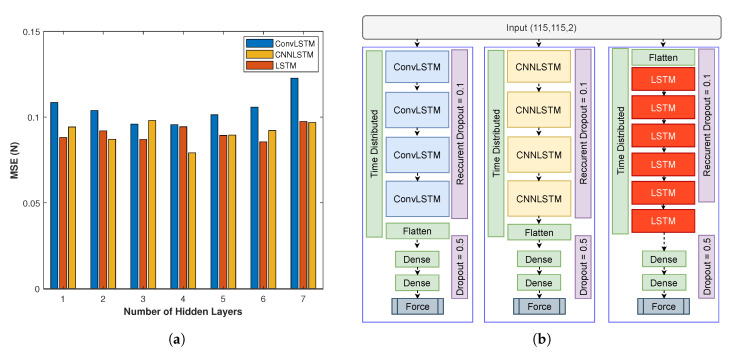
(**a**) Average MSE of Validation over all folds by varying number of hidden layers from 1 to 7 for ConvLSTM (Blue), LSTM (Red) and CNNLSTM (Orange). (**b**) Network architectures and configurations for ConvLSTM, CNNLSTM and LSTM.

The validation set is only used to tune hyper-parameters and select the optimal architecture. Afterwards, average of Mean Squared Error (MSE) over all folds is calculated on validation set to select the best hyper-parameters for each architecture. Figure 3a illustrates the average MSE over all folds for the validation set considering a range of 1 to 7 hidden layers. The minimum MSE is achieved with 4 hidden layers for ConvLSTM and CNNLSTM while LSTM network reaches the best performance with 6 hidden layers excluding the dense layers. The models run time is approximately 0.12 sec which can be further reduced with the speed optimization frameworks such as TensorRT. In average, the training time for a model takes 45 mins considering ConvLSTM architecture and 35 experiments. The training time depends on GPU model, number of experiments, and deep learning framework (Keras). Figure 3b presents networks architectures and configurations including dropout layers for the three dynamic methods.

After selecting a model with the lowest MSE for each architecture, average of MSE on a test set is calculated for evaluation purposes. Similar to [22], only non-zero measurements are considered to provide a realistic assessment of the sensor. Finally, we train and perform the same experiments for TDNN network to compare the proposed methods with our previous work [13]. Table 2 presents the average of Mean Absolute Error (MAE) and MSE over all the folds where the standard deviation is denoted as *σ*. The lowest errors are highlighted in bold.

**Figure 4:**
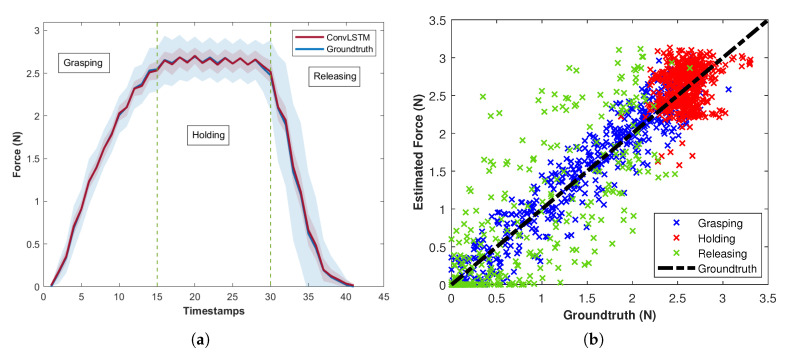
(**a**) Average of the estimated force and groundtruth for three phases over all folds. The highlighted area presents the standard deviation of values. (**b**) presents the scatter plot for estimated force in the grasping (blue), holding (red) and releasing (green) phases against the groundtruth (black line).

Although ConvLSTM validation error is higher than other models, this architecture generalizes better for the test set and achieves the highest accuracy considering all the phases. Figure 4a demonstrates the average of the estimated force and groundtruth over all folds during the grasping, holding and releasing phases Figure 4b demonstrates the estimated force values against the groundruth for different phases.

The main reason of a high standard deviation in groundtruth is the Baxter’s gripper vibration. Even though the experiments are repeated with the same configuration, the environment is not fully controlled to evaluate the sensor in a practical scenario. Besides the vibration, the object size also plays an important role in differences between experiments. Silicone material has a highly non-linear deformation in different phases due to the changes in the contact force and objects size. Most of research in the literature (see Table 1) considers only the loading phase to eliminate the impact of silicone elasticity from the measurement. Table 3 presents the average of MSE and standard deviation of error for each phase considering all folds.

The results indicate that the accuracy of the estimated force is very promising for the grasping phase whereas all three LSTM-based networks achieve similar results. Due to the elasticity of the silicone membrane, errors tend to be more significant during the holding and releasing phases. Also, the difference between accuracy of different network architectures increases continuously towards the end of releasing phase. Furthermore, the non-linearity of the silicone behaviourbehavior creates a variable time lag between the F/T sensor and the proposed sensor. In order to investigate this problem, we perform Dynamic Time Warping (DTW) and calculate the distance between the estimated force and groundtruth in different phases. To measure similarity of distributions between the estimated force and the F/T sensor, Bhattacharyya distance is considered for each phase of the grasp. Table 4 presents the average of Bhattacharyya distance ($D_{B}$) and DTW distance ($D_{W}$) over all folds for each phase of the grasp. Nevertheless, a slight time lag in the estimation leads to have a similar MSE in the holding phase for both CNNLSTM and ConvLSTM.

As presented in Table 4, both CNNLSTM and ConvLSTM achieve similar results considering $D_{W}$ and $D_{B}$ in the grasping phase. However, ConvLSTM achieves significantly lower $D_{B}$ during the holding phase. LSTM cells in CNNLSTM architecture perform on a vector which results in loss of spatial-temporal information while Convolutional LSTM (ConvLSTM) networks are more robust by keeping the input dimension inside LSTM layers. The low values of $D_{W}$ and $D_{B}$ for ConvLSTM in the holding phase indicate that it follows the contact force variations, caused by vibrations, better than other networks.

**Figure 5:**
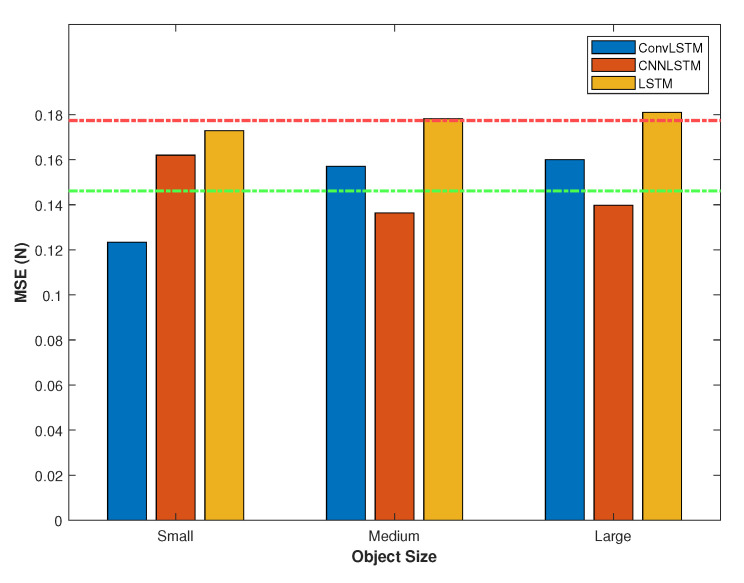
Average of MSE on all folds considering three LSTM-based networks for objects with different sizes. The green line shows average of MSE for ConvLSTM and CNNLSTM networks approximately. The red line illustrates the average of MSE for LSTM network.

In Figure 5, the averages of MSE for different sizes of bolts are illustrated. The ConvLSTM network achieves the highest accuracy for small objects while CNNLSTM performs better predictions for medium and large objects. The main reasons for various errors are limited data, random selection of experiments for the validation set, and non-linearity of silicone behaviourbehavior considering a larger contact area. The experimental setup is uncontrolled to evaluate the proposed method for practical applications. The experiment lengths are varied slightly for different objects due to the elasticity of the silicone membrane. Therefore, a slight difference in the contact force measurements distribution is recognizable in the experiments which affect accuracy of the network for different objects.

As demonstrated in Table 1, majority of the vision-based tactile sensors utilizuse static (without memory) DNNs without considering history of events in the contact area. In [22], Deep Neural Network (DNN) is considered with optical- flow inputs to estimate the contact force. The reported results (RMSE = 3 mN) are obtained in a controlled environment, where force values are obtained after the stabilization of a single object (intender). When a static DNN, such as the CNN-based transfer learning approach in [15] is applied in a variety of object sizes during the grasping phase, errors are much higher (RMSE = 1.2 N). In contrast, we proposed a dynamic approach to estimate the contact force continuously for objects with different sizes. Our approach provides a memory to the sensor in order to learn objects geometry at early stage of the grasping phase and adopt the contact force estimation during different phases. In the grasping phase, the proposed sensor achieves MSE = 0.064 N, comparable to state-of-the-art VBM sensors (Table 1), for objects with different sizes. Even for the holding phase where object vibration is irrefutable, a case that has not been considered by other VBM methods, MSE is only 0.082 N. Furthermore, we trained a new network (ConvLSTM 4 hidden layers) with small and large size objects and validated it on the experiments with the medium object. Twenty-eight experiments of experiments of large and small objects are used for training while twelve experiments of the medium size are considered for validation and testing (six for validation and six for the test set). The results indicate MSE of 0.159 N and MAE of 0.385 N on the test set during the all three phases. As expected, the error is higher but still acceptable when the sensor is used on objects with sizes different than the ones used for training.

## 7. Conclusions and Future Work

In this paper, we proposed a novel methodology to estimate the contact force dynamically. A neuromorphic camera (DVS) is used to capture intensity changes in the contact area. A novel dynamic approach is proposed to estimate the contact force for size-variant objects. The main challenge of force estimation for shape-variant objects is different contact area geometry under similar amount of applied force. We demonstrated that LSTM-based networks learn to relate the contact area size to the corresponding contact force over time.

Three LSTM-based networks are developed and implemented to estimate the contact force based on history of changes in every pixel considering both spatial and temporal features. The proposed sensor is validated on Baxter robot for three bolts with different sizes. ConvLSTM achieved the best results, specifically MSE = 0.064 N for estimating the contact force in the grasping phase and MSE = 0.082 N in the holding phase, despite the inevitable vibrations. The sensor has a low logical latency of 10 ms which is suitable for the real-time grasping applications. The main advantage of the proposed approach is the combination of convolutional networks with recurrent layers which enables the sensor to estimate the contact force based on the object size relatively. The ConvLSTM architecture learns objects geometry in early frames and estimate the contact force during a grasp.

For future work, synthetic data generation and augmentation techniques will be investigated to to increase the variability and the size of the training dataset and consequently increase the network generalization for the contact force measurements when an unknown object is grasped.

## Figures and Tables

**Table 1:** Comparison of state-of-the-art vision-based tactile sensor (σ represents standard deviation). The resolution of 3D force sensors are considered for the z-axis (normal force) measurements only.

Ref	Camera	Purpose	Method	Specifications
[22]	Frame-based	Force Distribution	Optical Flow DNN	Precise 3D force distribution Resolution of 0.003 N (MSE) Finite Element Method (FEM)
[17]	Frame-based	Force Measurement	Tracking Markers	3D force estimation Resolution of 0.29 N (MAE) Static approach
[15]	Frame-based	Force Measurement	CNN Transfer Learning	3D force estimation Resolution of 1.44 N (MSE) Static CNN transfer learning
[18]	Frame-based	Hardness Estimation	CNNLSTM	Shape-independent approach Unknown force and trajectory Dynamic CNNLSTM
[24]	Frame-based	Force Distribution	Marker Tracking	Range of 3–4 N *σ* = 0.322 N for normal force Known object geometry
[25]	DVS	Incipient Slip Detection	Morphological Operations	Latency of 44.1 ms Shape and material independent Traditional image processing
[26]	DVS	Incipient Slip Detection	Image Analysis	Slip detection Event-framing over 500 μs Orientation estimation
[13]	DVS	Force Estimation	TDNN and GP	Logical latency 21 ms Resolution of 0.16 N (MSE) Different materials
This Work	DVS	Force Estimation	LSTM-Based Networks	Dynamic approach Logical latency of 10 ms Resolution of 0.064 N (MSE) Size-independent

**Table 2:** Average error of the estimated force and standard deviation (σ) for the test set.

Network/Errors	MAE(*σ*)	MSE(*σ*)
TDNN	0.398(0.410)	0.345(0.713)
LSTM	0.301(0.234)	0.160(0.261)
CNNLSTM	0.291(0.234)	0.157(0.259)
**ConvLSTM**	**0.278(0.225)**	**0.145(0.237)**

**Table 3:** Average of MSE (N) and standard deviation (σ) of the estimated force over all folds for unseen experiments. The lowest errors are presented in bold.

Phase	Grasping		Holding		Releasing	
Methods	MSE	*σ*	MSE	*σ*	MSE	*σ*
TDNN	0.309	0.572	0.190	0.426	0.490	0.425
LSTM	0.065	0.053	0.092	0.065	0.537	0.407
CNNLSTM	**0.063**	**0.051**	0.088	0.064	0.527	0.386
ConvLSTM	0.064	0.055	**0.082**	**0.063**	**0.485**	**0.372**

**Table 4:** Comparison of Dynamic TimeWarping Distance (*D_W_*) and Bhattacharyya Distance (*D_B_*) for three different networks architectures. The lowest values are presented in bold.

Phase	Grasping		Holding		Releasing	
Methods	*D_B_*	*D_W_*	*D_B_*	*D_W_*	*D_B_*	*D_W_*
LSTM	0.102	1.711	2.278	3.811	0.622	2.282
CNNLSTM	**0.009**	**1.642**	2.483	3.588	0.061	**2.204**
ConvLSTM	0.009	1.678	**1.175**	**3.258**	**0.055**	2.346
